# Can bile duct injuries be prevented? "A new technique in laparoscopic cholecystectomy"

**DOI:** 10.1186/1471-2482-5-14

**Published:** 2005-06-17

**Authors:** Yavuz Selim Sari, Vahit Tunali, Kamer Tomaoglu, Binnur Karagöz, Ayhan Güneyİ, İbrahim KaragöZ

**Affiliations:** 1 SSK İstanbul Training Hospital Department of General Surgery – Istanbul, Turkey; 2 Saint Georg Hospital Department of General Surgery, Hamburg, Austria

## Abstract

**Background:**

Over the last decade, laparoscopic cholecystectomy has gained worldwide acceptance and considered to be as "gold standard" in the surgical management of symptomatic cholecystolithiasis. However, the incidence of bile duct injury in laparoscopic cholecystectomy is still two times greater compared to classic open surgery. The development of bile duct injury may result in biliary cirrhosis and increase in mortality rates. The mostly blamed causitive factor is the misidentification of the anatomy, especially by a surgeon who is at the beginning of his learning curve. Biliary tree injuries may be decreased by direct coloration of the cystic duct, ductus choledochus and even the gall bladder.

**Methods:**

gall bladder fundus was punctured by Veress needle and all the bile was aspirated. The same amount of fifty percent methylene blue diluted by saline solution was injected into the gall bladder for coloration of biliary tree. The dissection of Calot triangle was much more safely performed after obtention of coloration of the gall bladder, cystic duct and choledocus.

**Results:**

Between October 2003 and December 2004, overall 46 patients (of which 9 males) with a mean age of 47 (between 24 and 74) underwent laparoscopic cholecystectomy with methylene blue injection technique. The diagnosis of chronic cholecystitis (the thickness of the gall bladder wall was normal) confirmed by pre-operative abdominal ultrasonography in all patients. The diameters of the stones were greater than 1 centimeter in 32 patients and calcula of various sizes being smaller than 1 cm. were documented in 13 cases. One patient was operated for gall bladder polyp (our first case). Successful coloration of the gall bladder, cystic duct and ductus choledochus was possible in 43 patients, whereas only the gall bladder and proximal cystic duct were visualised in 3 cases. In these cases, ductus choledochus visibility was not possible. None of the patients developed bile duct injury.

**Conclusion:**

The number of bile duct injuries related to anatomic misidentification can be decreased and even vanished by using intraoperative methylene blue injection technique into the gall bladder fundus intraoperatively.

## Background

Laparoscopic cholecystectomy (LC) is considered as the "golden standard" in the surgical management of symptomatic cholelithiasis. Short hospitalisation period and rapid return to normal activity, less post-operative pain, more acceptable cosmetic results and lesser morbidity and mortality rates, are the principle advantages of this technique. However, the incidence of bile duct injuries is two times greater when compared to open cholecystectomy [1-11]. Bile duct injury, either in classic open or laparoscopic cholecystectomy, may necessitate several consecutive operations and invasive procedures, causing fear and anxiety to all surgeons.

The development of bile duct injuries following LC is not common but a serious complication resulting in long-term morbidity [8,12]. When the literature is reviewed, the incidence of bile duct injuries in LC is between 0,3 – 0,6 % [4,6-10,13-15], which may be considered an acceptable percentage, may in fact result in secondary biliary cirrhosis with considerable financial burden [6,8,10]. Higher incidence of biliary tree injuries has also been reported [3]. In United States, 600 000 cases of laparoscopic cholecystectomies are performed annually. When this number is taken into consideration, it will be clearly understood that the economic problem caused by even small (0,3 – 0,6 %) rates of bile duct injuries, can not be underestimated[1,15].

Herein, we introduce a new technique, with the hope to reduce bile duct injuries during LC and we publish the results of 46 cases.

## Methods

The patients were installed in French position. The trocards were placed as in French position. The gall bladder fundus was grasped and held tight towards the anterior abdominal wall with the help of two atraumatic pinces (or graspers) introduced via right anterior axillary and subxyphoid trocards. The gall bladder fundus was punctured by a Veress needle which was introduced via the abdominal wall in projection to this area.

**Figure 1:**
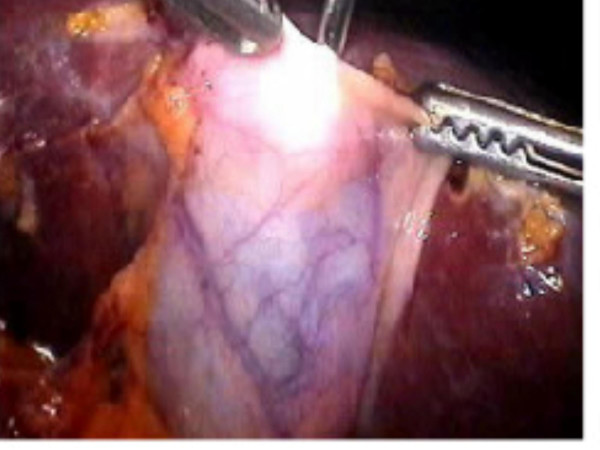
The injection of methylene blue into the gall bladder.

**Figure 2:**
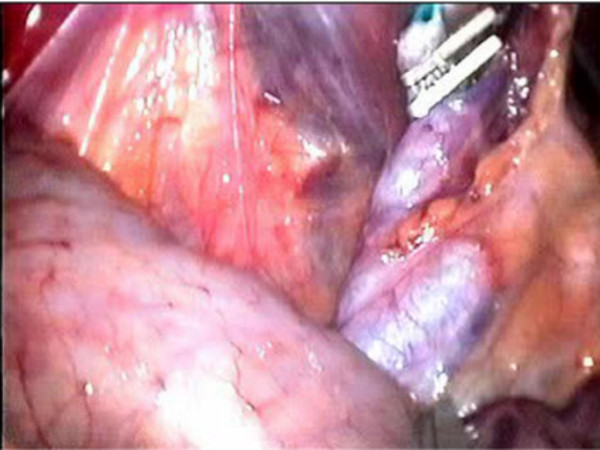
The visualisation of cystic duct and ductus choledochus with methylene blue after removal of gall bladder.

All the bile in the gall bladder was aspirated and 50 percent diluted methylene blue equal to the amount of aspirated bile was injected slowly into the gall bladder (Figure 1). In order to prevent bile leakage, the gall bladder fundus was held tight anteriorly during the withdrawal of the Veress needle and a grasper introduced via the xyphoid trocard was applied immediately to the puncture site and was held so throughout the operation. During cholecystectomy, the gall bladder, cystic duct and ductus choledochus were visible with methylene blue dye and the dissection was performed more safely (Figure 2).

The gall bladder was removed from the abdominal cavity through the trocar inserted from lateral border of left rectus muscle. In order to minimize bile leakage into the abdominal cavity, the gall bladder was completely aspirated before removal from the abdominal cavity.

## Results

Between October 2003 and December 2004, 46 patients underwent LC by "Methylene blue dye injection" technique. 37 patients were female (mean age 45) and 9 patients were male (mean age 52). Chronic cholecystitis was found in all patients in pre-operative ultrasonographic evaluation. (wall thickness of the gall bladder was normal). The diameter of the stones in 32 patients was more than 1 centimeter and multiple small stones were found in 13 patients. One patient was operated with the diagnosis of gall bladder polyp (first case). The gall bladder, cystic duct and ductus choledochus were painted with methylene blue in 43 cases but only the gall bladder and the proximal cystic duct were visualised in 3 cases.

In 5 cases operated by the residents, methylene blue leakage from the gall bladder was observed into the abdominal cavity during the removal procedure. The region was irrigated with saline solution. All patients were informed that they might pass blue urine in the early post-operative period. None of the patients developed any complication and all of them were discharged the day after the operation. We did not use this technique in acute cases.

In one case, the trajectory of the cystic duct was very close to the right hepatic duct and its opening to main hepatic duct was just distal to the bifurcation. A trifurcation was demonstrated in one case where the cystic duct was directly opening to the branch of 6^th ^and 7^th ^hepatic segments. These anatomic variations were clearly demonstrated with our technique.

## Discussion

Many factors have been incriminated in occurance of bile duct injuries during LC. These are mainly anatomical misidentification of main hepatic duct, right hepatic ducts or of aberrant right hepatic duct as ductus cysticus, other anatomical variations or unidentifiable anatomy, surgeon's experience (a surgeon who is at the beginning of his learning curve), technical difficulties, poor visualization of the operative field, acute and chronic inflammation of the gall bladder and local factors such as excessive haemorrage and fat tissue [1-3,5-8,15-17]. On the other hand, the problems related to the equipment have been accused [8,9]. However, misidentification of the anatomy and surgeon's experience seem to be preliminary [1,3,5,9,11,12,14-17].

Bile duct injuries are associated with significant morbidity, prolonged hospitalization, increased financial burden, potential litigation and occasional mortality [1,5,6,10,11]. It is the third most commonly litigated general surgical complication in The United States and it has been also reported that on the average two procedures (between 1 to 8) are required for definitive repair of bile ducts [5]. Obviously, if bile duct injury is noticed peroperatively and repaired in the best way, morbidity and mortality rates would be significantly reduced.

Although the importance of intraoperative cholangiography (IOC) to prevent bile duct injury is stressed by a significant number of authors, its role still remains controversial [1,5,8,9,15,17,18]. Despite its potential benefits, routine use of IOC has not taken its place in the surgical era. [2,4,5]. It has been claimed that routine use of IOC does not have a significant practical advantage [1,2,5,15,17]. Additionally, the operation room conditions should be suitable for IOC. Other disadvantages of IOC are; the necessity of some disposable equipment, the need of surgical experience, the inevitable prolongation of the operation time and the need of interpretation by an experienced radiologist.

During medical education, in Textbooks of Anatomy, we have seen the arteries nicely colored in red, the veins in blue and the lymphatics in yellow. Later on, facing the truth in cadavers, we were all somewhat disappointed. The idea of using methylene blue dye intraoperatively to colorise the anatomic details, is in fact based on this simple truth. The basic principle is to minimize the probable injuries by painting the gall bladder, ductus cysticus and ductus choledochus peroperatively.

As explained in the technique, a few minutes after methylene blue injection into the gall bladder, the gall bladder, ductus cysticus, ductus choledochus and in most of the cases even the duodenum have been painted. So the dissection can easily be performed. The operation time has not been significantly increased by methylene blue injection. Also, it is not associated with increased cost. Additionally, the flow of methylene blue from the nasogastric tube (noted by the anaesthesiologist) and/or the coloration of the duodenum may lead to indirect conclusion that the bile duct flow is uninterrupted.

When the images obtained by IOC are to be evaluated in the operating room, with this technique, a comparison with the anatomic details of the operating field may also be possible.

No bile duct injuries were encountered in 46 cases. This result may certainly not be directly attributable to our technique, however, it is true that the dissection was performed much more safely. Since the boundaries of the gall bladder were significantly painted with methylene blue, the residents managed to remove the gall bladder from the liver bed without causing any perforation.

Obviously, overlooked bile duct injuries during LC appears to be another problem [6,12-14,19]. We believe that it is possible to notice the bile duct injury with this technique because of methylene blue leakage from the injured area. In this case, the decision for conversion to open surgery may be easily taken into consideration.

Because methylene blue is excreted by the kidneys, the patients should be informed of the possibility of blue urine in the early post-operative period.

LC is successfully used in acute cases [7,20]. We did not use this technique in acute cases.

## Conclusion

Bile duct injury during cholecystectomy is always a possibility, no matter which technique is used. In fact, the incidence of bile duct injury during laparoscopic cholecystectomy is slightly elevated compared to classic open surgery. A bile duct injury may be the beginninig of a catastrophic sequence of a serious complication. We believe that the incidence of bile duct injury related to anatomic misidentification can be decreased or even totally suppressed by intraoperative injection of methylene blue into the gall bladder fundus and visualisation of the gall bladder, cystic duct and ductus choledochus.

## Pre-publication history

The pre-publication history for this paper can be accessed here:


